# Sampling Capacity Underlies Individual Differences in Human Associative Learning

**DOI:** 10.1037/xan0000012

**Published:** 2014-01-20

**Authors:** Nicola C. Byrom, Robin A. Murphy

**Affiliations:** 1 Department of Experimental Psychology, University of Oxford, Oxford, England

**Keywords:** individual differences, global processing, associative processes, generalization

## Abstract

Though much work has studied how external factors, such as stimulus properties, influence generalization of associative strength, there has been limited exploration of the influence that internal dispositions may contribute to stimulus processing. Here we report 2 studies using a modified negative patterning discrimination to test the relationship between global processing and generalization. Global processing was associated with stronger negative patterning discrimination, indicative of limited generalization between distinct stimulus compounds and their constituent elements. In Experiment 2, participants pretrained to adopt global processing similarly showed strong negative patterning discrimination. These results demonstrate considerable individual difference in capacity to engage in negative patterning discrimination and suggest that the tendency toward global processing may be one factor explaining this variability. The need for models of learning to account for this variability in learning is discussed.

Generalization of associative strength is a fundamental mechanism by which previous experience might guide expectation in novel situations. Most theories of learning assume that generalization of associative strength occurs as a function of stimulus similarity (Bush & Mosteller, 1951; McLaren & Mackintosh, 2002; Pearce, 1987; Rescorla, 1976; Rescorla & Furrow, 1977). Because, in principle, every stimulus is unique, stimuli may be conceived as composed of multiple elements, some of which will be common to other stimuli whereas others may be unique. In this way, similarity is quantified as the proportion of elements that stimuli share in common and generalization is predicted to occur to the extent that stimuli share common features (Kruschke, 1992; Mackintosh, 1975; McClelland & Rumelhart, 1985; Nosofsky, 1984; Pearce, 1987; Rescorla & Wagner, 1972; Van Hamme & Wasserman, 1994). In this paper we examine whether generalization of learned associations between stimuli is related to the perception of stimulus elements as independent from their whole and whether such perception is modifiable with relatively short training.

This question builds on the wealth of research comparing elemental and configural models of learning. Elemental models (e.g., Brandon, Vogel, & Wagner, 2000; Estes, 1950; Rescorla & Wagner, 1972) assume that the representation of individual elements enter into independent associations with outcomes and might summate to determine expectation. Configural models (e.g., Pearce, 1987) assume that it is a configuration of these stimuli that enters into association with outcomes. These models make substantively different predictions about outcome expectation when stimuli are combined. For instance, if two stimuli are associated with a particular outcome (i.e., A+, B+) a range of outcomes may be expected following the co-occurrence of these stimuli (i.e., AB). The elemental approach of linearly summing the outcome associated with each separate stimulus predicts that double the outcome should be expected following the compound (AB++; Rescorla & Coldwell, 1995; Rescorla & Wagner, 1972). In contrast a configural model (Pearce, 1987, 1994) assumes that outcome expectation following the compound will be determined on the basis of similarity between the stimuli and compound; as AB is 50% similar to A and 50% similar to B, the expectation of outcome following the AB compound should be 50% of that following A plus 50% of that following B (AB+). Though there have been many comparisons of the two models (e.g., Vallée-Tourangeau, Murphy, & Baker, 1998), such comparisons have not provided conclusive support for either model. Instead, the data collected from such comparisons suggests that it may be more productive to accept that elemental and configural patterns of learning can occur (Fanselow, 1999; Melchers, Shanks, & Lachnit, 2008; Williams, Sagness, & McPhee, 1994, but see Livesey & Harris, 2008; McLaren, 2008). Our task then should be to understand when learning should reflect an elemental model and when it should reflect a configural model, and which factors elicit such a change.

Tasks such as the negative patterning discrimination have been widely used to compare elemental and configural predictions. The negative patterning discrimination requires participants to learn that combinations of stimuli are predictive of outcomes independent from the outcomes associated with their constituent stimuli; in the same way that a face is a distinct configural whole, different from constituent elements (eyes, nose, etc.). As such, the task provides a tool to test the ability to learn about configurations of stimuli. One modified version of the negative patterning task (A+/B+/C+, AB+/AC+/BC+, ABC–) has been used extensively to compare and contrast elemental and configural predictions of learning (e.g., Kinder & Lachnit, 2003; Redhead, 2007; Redhead & Pearce, 1995). For this discrimination, elemental models predict that learning should occur more readily with the double compounds than the single stimuli, while configural models predict that discrimination learning should occur more readily with the single stimuli than the double compounds (Redhead & Pearce, 1995).

Testing these predictions, Redhead (2007) found that human participants learned this discrimination both when learning about unimodal stimuli (Experiment 1) and multimodal stimuli (Experiment 2). In both experiments participants’ ratings for the single stimuli was significantly higher than their ratings for the double compounds, indicating better discrimination with the single stimuli than the double compounds. Kinder and Lachnit (2003) used a similar design to compare positive (D–/E–/F–, DE–/DF–/EF–, DEF+) and negative (A+/B+/C+, AB+/AC+/BC+, ABC–) patterning as human participants learned about unimodal stimuli. They observed a different pattern of results; though participants learned the negative patterning discrimination, no differences between ratings for the single stimuli and double compounds were observed. The contrasting findings of these two studies reflect earlier observations in studies with animals (Myers, Vogel, Shin, & Wagner, 2001; Redhead & Pearce, 1995). Taken together these studies have not provided conclusive support for either an elemental or a configural model of learning. Both models have trouble accounting for the variety of observed results.

Reviewing these and many other experiments, Wagner introduced a replacement parameter to the replaced elements model (REM) of learning and concluded that “a major determinant of the replacement parameter, r, is the different stimuli employed” (Wagner, 2003, p. 26). REM conceives of stimuli as represented by multiple elements, in which a proportion of the elements represented change with the context within which the stimulus is presented (Brandon et al., 2000). Wagner (2003) argued that when training occurs with multimodal stimuli, *r* can be assumed to be small, so that when stimuli are presented in compound few elements should be replaced. In converse, *r* could be assumed to be high when stimuli are from the same modality, such that perceptual interactions are likely. Kinder and Lachnit (2003) introduced a discriminability parameter to Pearce’s (1987) configural model of learning to achieve a similar effect. The discriminability parameter assumes that the perceived similarity between configurations may change. The discriminability parameter decreases as it becomes harder to identify constituent stimuli within a configuration. This may be likely to happen if perceptual interaction occurs (Kinder & Lachnit, 2003).

Following these approaches to flexibility between elemental and configural learning, it may appear that changes in the properties of stimuli are the primary factor influencing variation in learning. Several studies however have observed variation in human learning without changing the stimulus properties. For instance, Williams and Braker (1999) observed that a participant’s learning depended on whether he or she had received pretraining with a task requiring an elemental or configural solution. Williams, Sagness, and McPhee (1994) similarly observed blocking after providing elemental but not configural pretraining. Blocking occurs when training a stimulus (A) to predict an outcome prior to training a compound (AB) to predict the same outcome, prevents the second stimulus (B) from acquiring associative strength. Williams et al. (1994) suggested that if participants used a configural strategy, they attributed the outcome to the configuration rather than the separable predictive stimuli, preventing the competition effect between the two stimuli (A and B) that produces blocking. Williams et al. (1994) suggested that the prior learning may have influenced subsequent strategy in one of two ways; prior learning may change the extent to which attention is directed toward elements or compounds or may alter the perceived similarity between elements and compounds. As these studies use associative learning training to influence subsequent learning, it may be argued that prior training teaches participants a form of rule that they apply to subsequent discriminations.

Rule use has been observed to facilitate performance in a negative patterning task, suggesting that ability to acquire and use a rule may influence learning strategy. Using a combination of negative and positive patterning (A+, B+, AB–; C–, D–, CD+), Shanks and Darby (1998) found differences between individuals that may relate to ability to engage rule based learning. In particular, they observed an association between good discrimination learning and later rule based generalization. Recent studies have suggested that capacity to attend to the task may account for some of the observed variation in configural learning (Juslin, Olsson, & Olsson, 2003; Shanks & Darby, 1998; Wills, Graham, Koh, McLaren, & Rolland, 2011). For instance, Wills et al. (2011) found that participants completing this task under full attention were more likely to adopt rule based generalization than participants who completed a concurrent task while learning the negative patterning discrimination. As yet however the factors that influence individual differences in human configural learning remain unclear.

Accounts of variation in learning on the basis of the perceptual properties of stimuli have focused on the effect of perceptual interaction (e.g., Kinder & Lachnit, 2003; Wagner, 2003). This can be conceptualized as variation in the extent to which elements of a stimulus sampled change depending on the context in which the stimulus is represented. The replacement parameter in the REM does this explicitly, by varying the proportion of elements replaced. Although the impact of relative variation in sampling has been considered extensively, it is conceivable that, under certain conditions the absolute sampling capacity itself may vary. Individuals might differ in terms of the number of stimulus features they sample simultaneously, or the same individual might increase or decrease the number of items sampled, through practice, attention, or other motivational or dispositional factors. In this paper we test whether differences in ability to learn a configural discrimination occur independent from changes in the perceptual properties of the stimuli and explore whether differences in absolute sampling capacity may provide a mechanism for variation in learning. In particular we test whether the tendency to attend to configural wholes as opposed to local details influences configural learning.

Individuals differ in their tendency to focus on global or local information (Navon, 1977). Global processing is associated with better recognition of faces (Macrae & Lewis, 2002; Perfect, 2003), a task thought to be highly sensitive to configural processing (Diamond & Carey, 1986; Leder & Bruce, 2000; Maurer, Le Grand, & Mondloch, 2002; Tanaka & Farah, 1993). The question of whether an individual focuses on the global stimulus is of particular interest as low mood and high stress have been associated with a tendency to focus on local details (Basso, Schefft, Ris, & Dember, 1996; Fredrickson & Branigan, 2003; Gasper & Clore, 2002). Individual difference in the tendency to attend to local or global information can be measured using the Navon (1977) task. The Navon task tests the participant’s tendency to focus on global information relative to the specific elements (local information) composing global figures. In this task, participants are presented a large letter (the global stimulus) constructed out of small letters (the local stimulus); for example, a large *H* consisting of small *H*s. The letters are either congruent, where the local and global stimuli are the same or incongruent, where the local and global stimuli differ. On different trial blocks, participants are asked to report the global or local stimulus. Navon found that, in general, participants responded faster for the global stimulus than the local stimulus; this has been termed the global precedence effect (Navon, 1977). Using the Navon task enables us to test whether differences in ability to identify global targets relate to differences in configural learning. Individual difference in ability to identify global targets may reflect differences in a common configural system that underlies the perception of global stimuli and the ability to configure stimuli during learning.

In the experiments presented here, participants completed a modified negative patterning task intermixed with a linear discrimination. As global processing has been associated with ability to encode configurations (e.g., Macrae & Lewis, 2002; Perfect, 2003), individuals showing a global processing advantage may be expected to show strong learning in the negative patterning discrimination. We hypothesized that global processing may reflect a broad sampling capacity and as such might facilitate learning about configurations and will therefore be associated with strong negative patterning discrimination. In contrast, a local processing advantage may reflect a narrow sampling capacity and as such result in weak negative patterning discrimination. As learning a linear discrimination depends only on the one-to-one correspondence between separate stimuli and the outcomes they predict, a local processing advantage should have no impact on ability to learn a linear discrimination.

## Experiment 1

A modified version of the negative patterning task developed by Redhead and Pearce (1995; A+/B+/C+, AB+/AC+/BC+, ABC–) was used. In this experiment participants were required to learn that a single stimulus (A+) and a double compound (BC+) predicted one outcome but the configuration of all three stimuli (ABC–) predicted an absence of outcome. As each separate stimulus was paired with outcome as frequently as it was paired with the absence of an outcome, solving this discrimination depended on learning about configurations of stimuli as opposed to learning about the separate stimuli. Ability to learn this discrimination was compared to ability to learn a linear discrimination, in which a single stimulus (D–) and a double compound (EF–) were paired with no outcome while a triple compound (GHI+) was paired with the outcome. More important, the compounds in this linear discrimination did not share any stimuli in common and the discrimination could be acquired without learning about the configurations of the co-occurring stimuli.

Participants’ response time to incongruent stimuli in the Navon task were used to provide an indication of whether participants focused more readily on the global or local stimuli. Participants focusing on global stimuli should respond faster on incongruent trials when asked to identify the global cue. Participants focusing on the local stimuli will respond faster on incongruent trials when asked to identify the local cue. As the global and local stimuli are the same on congruent trials, these trials were not used to calculate the tendency toward local or global processing. This is because participants can respond to the global stimulus in a block of trials where they have been asked to identify the local stimuli and their response will appear correct. Congruent trials however were included in the test procedure to ensure that participants were not using an explicit rule, such as the local stimulus is always the opposite of the global stimulus.

### Method

#### Participants

There were 41 university students who participated for course credit or were paid £5 for their participation. Thirty-two of the participants were female. The average age was 20.32 years (*SEM* = 0.40) years and average working memory capacity, as tested by digit span (Lezak, 1995) was 7.50 (*SEM* = 0.11) digits.

#### Stimuli and materials

##### Assessment measures

Participants completed the digit span assessment of working memory capacity (Lezak, 1995). The Beck Depression Inventory (BDI; Beck, Ward, Mendelson, & Erbaugh, 1961) and the Neuroticism subscale of the Neuroticism, Extraversion, and Openness Personality Inventory Revised (NEO–PI–R; Costa & McCrae, 1995) were completed to provide a measure of depressive state and trait risk for depression. These questionnaires were administered at the start of the experiment to ensure that the experience of the learning and attention tasks did not influence how participants completed the questionnaires.

##### Negative patterning

**Figure 1 fig1:**
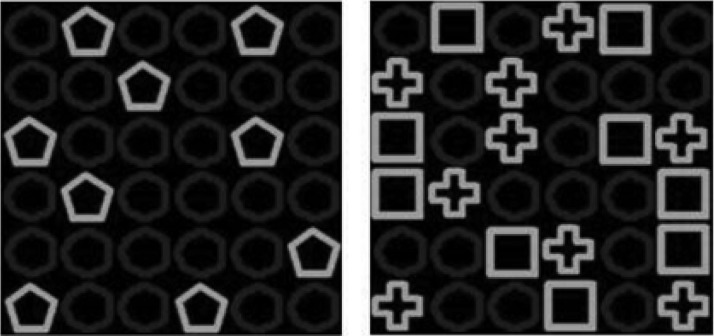
Stimuli used in the negative patterning tasks in Experiment 1 and Experiment 2. Left: stimulus presentation on a single stimulus trial (i.e., A); right: stimulus presentation on a double stimulus compound trial (i.e., BC).

The learning task was embedded in a cover story describing bacteria and cell growth. The stimuli, as shown in Figure 1, were nine brightly colored shapes presented in squares on a black grid of 6 × 6 squares, measuring a total of 52 mm × 52 mm. The shapes (measuring 8.50 mm × 8.50 mm) were: upright triangle, upside down triangle, square, circle, kite, pentagon, diagonal, and cross. The remaining squares in the grid were filled with darkly colored circles. Assignment of shape to experimental stimulus was partially counterbalanced, such that each shape was assigned to a different stimulus position giving nine configurations. The occurrence of outcome was shown by cells growing to cover the computer screen.[Fig-anchor fig1]

##### Navon task

**Figure 2 fig2:**
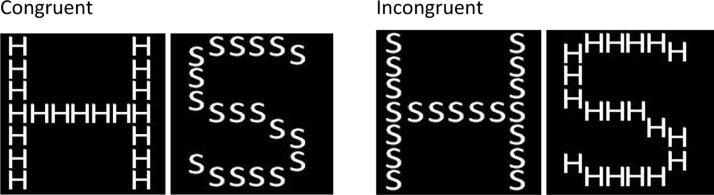
Stimuli used in the Navon task, showing congruent (left) and incongruent (right) stimuli.

The Navon task, presented on computer (programmed in Visual Basic, Microsoft), used four separate stimuli, shown in Figure 2. All stimuli consisted of white letters presented on a black background. Stimuli were presented in a square with the large letters spanning 55 mm × 55 mm (6.30° × 6.30°) and the small letters spanning approximately 5 mm × 5 mm (0.60° × 0.60°). A stimulus size, toward the upper boundary of that identified as capable of inducing a global precedence effect (Kinchla & Wolfe, 1979) was used to allow for a reasonable likelihood of observing individual difference in global tendency. If stimuli are particularly small, all participants might be expected to show a global advantage. With very large stimuli, all participants might be expected to show a local advantage.[Fig-anchor fig2]

#### Procedure

Participants completed the working memory task and questionnaires prior to the learning task and then the Navon task. The Navon task was completed after the learning discrimination to ensure that the experience of completing the Navon task did not influence performance on the learning task.

##### Learning task

Participants were asked to imagine themselves as a biologist in a virtual laboratory. They were informed that they would be required to learn which bacteria caused cell growth. They were informed that they would see bacteria on multiple occasions and that while on the first presentation they would have to guess how much cell growth the bacteria caused, the relationship would become clearer as the task proceeded.

Participants discriminated between six stimuli compounds; three events in the negative patterning discrimination (A+, BC+, ABC–) and three separate events in the linear discrimination (D–, EF–, GHI+). Participants completed 14 blocks of six trial types. Trial order was randomized within blocks. On each trial, a stimulus or stimulus compound was presented in the center of the screen. Using a scale of 1 to 9 on the keyboard, participants made a rating of the likelihood that the presented stimulus would cause cell growth, where 9 represented *cell growth* and 1 represented *no cell growth*. Participants were instructed to use the scale as follows; “If you think that the bacteria does not promote much, or any, cell growth, select numbers toward the bottom of the scale. If you think the bacteria promotes cell growth, select numbers toward the top of the scale.”

Following the participants’ response, the outcome was presented. For outcome present trials, cell growth covered more than half of the screen, varying at random between 70%, 80%, 90%, and 100%. For outcome absent trials, cells covered less than half of the screen, varying at random between 0%, 10%, 20%, and 30%. Random variation within each bracket of growth was introduced to maintain participants’ engagement with the task. The outcome was presented for 2.50 s, followed by the opportunity to start the next trial.

The linear discrimination provided a measure of a participant’s ability to understand the basic requirements of the learning task. If a participant was unable to understand the linear discrimination, interpretation of their performance in the negative patterning discrimination would not be possible. At the end of training one individual continued to rate stimuli paired with no outcome as more predictive of the outcome than stimuli paired with outcome. This individual was excluded from further analysis.

##### Navon task

Participants were informed that they would be presented with a series of large letters composed of small letters and on successive blocks they would be asked to identify the large letter or the small letter presented. Participants were warned that the letters would be presented for a very short period of time.

Participants completed eight blocks of 16 trials. Each trial block contained eight *S* stimuli and eight *H* stimuli. Half the stimuli were congruent, half incongruent. The order of stimuli presentation was randomized within each block. On half of the trial blocks participants identified the small letters, on the other half the large letters. Trial blocks alternated and half of the participants started by identifying the large letters whereas half started by identifying the small letters.

On each trial a fixation point was presented in the center of the screen for 500 ms. This was followed by a stimulus presented for 40 ms. A mask replaced the stimulus and remained on the screen until participants made a response using the *S* or *H* using keys on the keyboard. Following their response there was a 300-ms intertrial interval. Response time, measured from stimulus onset and response accuracy were recorded.

### Results and Discussion

#### Navon task

Response times and response accuracy on the Navon task were recorded for four different trial types; global congruent, global incongruent, local congruent, and local incongruent. Average response accuracy was 87.50%. Only response times on correct trials were analyzed (Navon, 1977). To ensure that differences in response times between the trial types were not influenced by participants responding before they had seen the stimulus or some general lack of attention, outlying responses for each trial type, for each participant were removed from the analysis (Ratcliff, 1993). Response times that were greater than one standard deviation above or below the mean for that trial type were removed. On average four responses were removed for each trial type.

Response times on global incongruent trials were subtracted from response times on local incongruent trials to give a global processing score, such that higher scores reflected an advantage identifying targets on global trials. The mean global processing score was 3.26 (*SEM* = 1.20). A median split of global processing score was used to categorize participants into a local (*N* = 20) or global (*N* = 20) group. There were no significant differences between these groups in response accuracy, *t*(37) < 1, *p* = .50, working memory capacity, *t*(37) < 1, *p* = .90, age, *t*(37) < 1, *p* = .58, or gender, *t*(37) < 1, *p* = .49. We used a median split to allow detailed repeated-measures analysis of learning data. We also use the full continuum of global processing score in a regression analysis.

The BDI and a measure of Neuroticism were taken to explore whether personality or depressive state related to individual difference in performance on the Navon task, and if they did, whether this relationship mediated the interaction between global processing and learning. Neither Neuroticism, *r*(40) = −.10, *p* = .53, nor depressive state *r*(40) = −.06, *p* = .73 correlated with global processing score. As no significant correlations were observed, Neuroticism and BDI were not included in further analysis.

#### Learning task

Participants’ judgments of the probability that cell growth would follow a stimulus or stimuli compound were recorded. Average judgment over the first two trials were used for the initial trial block, average judgments over four consecutive trials were used to calculate Trial Blocks 2, 3, and 4.

**Figure 3 fig3:**
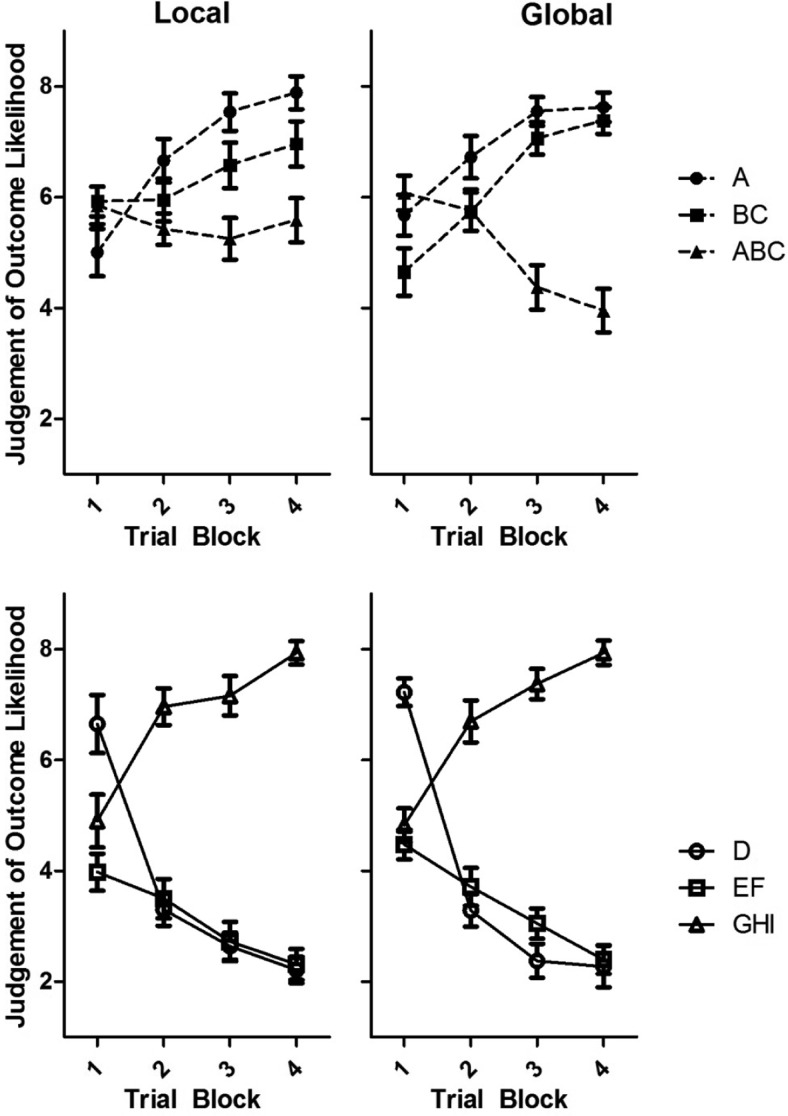
Judgments of outcome likelihood over trial blocks for Experiment 1, showing the local group (left) and the global group (right), for the negative patterning discrimination (top) and linear discrimination (bottom).

Judgments of outcome likelihood over training are shown in Figure 3. This analysis addresses whether participants differed in their ability to acquire a configural discrimination: the negative patterning discrimination. Analysis of judgments of outcome likelihood following the two compound stimuli in the negative patterning discrimination, BC and ABC, provides a simple assessment of differences in such ability. These compounds share two stimuli in common and predict different outcomes, placing a requirement on participants to learn about the combinations of stimuli presented in the compound. Ability to acquire the discrimination between BC and ABC was compared to ability acquiring the discrimination between EF and GHI. To learn the discrimination between EF and GHI, there is no requirement to learn about the combination of stimuli presented in the compound.[Fig-anchor fig3]

#### Differences in judgments for single stimuli and double compounds

Learning about the single stimulus A was not used to investigate the central hypothesis although we did find that at the end of training participants judged A as more likely to be followed by the outcome than BC, consistent with predictions of a configural models (e.g., Pearce, 1987). As shown in Figure 3, at the start of training participants did not differ in their judgments of outcome likelihood following A compared to BC, *t*(39) < 1, *p* = .91. This was the case for both the local, *t*(19) = 1.53, *p* = .14 and the global, *t*(19) = 1.83, *p* = .08 groups. At the end of training, participants judged A as somewhat more likely to be followed by the outcome than BC, *t*(39) = 2.11, *p* = .05, *d* = 0.42, 95% CI [0.02, 1.14]. However, split by group, neither the local, *t*(19) = 1.53, *p* = .14, nor the global, *t*(19) = 1.98, *p* = .06 group showed a statistically significant difference in judgments of A and BC. Independent samples *t* tests for the last trial block of training revealed that the two participant groups did not differ in their judgments of outcome likelihood following A, *t*(38) < 1, *p* = .52 or BC, *t*(38) < 1, *p* = .38. As we have not used learning with A to investigate our central hypothesis, it should be noted that, on average, all participants learned the discrimination between A and ABC better than they learned the discrimination between BC and ABC and, at the end of training, all participants gave judgments of outcome likelihood of above five (average) following A. Only four participants gave higher judgments of outcome likelihood following BC than A. This should provide confidence that participants, acquiring the BC-ABC discrimination, did not fail to acquire the A–ABC discrimination.

#### Differences in discrimination learning

To address group differences in discrimination learning, analysis focused on the discrimination between double and triple compounds. A four way repeated-measures analysis of variance (ANOVA) was conducted on judgments of the outcome likelihood with the factors of outcome (cell growth vs. no cell growth), discrimination (linear vs. negative patterning), trial block (first vs. last), and group (local vs. global). This analysis revealed a significant four way interaction, *F*(1, 38) = 8.20, *MSE* = 1.44, *p* < .01, η_p_^2^ = 0.18). To understand this interaction analyses of judgments of outcome likelihood, with the factors of discrimination (linear vs. negative patterning), outcome (cell growth vs. no cell growth), and group (local vs. global), have been conducted for the first and last trial block.

On the first trial block of training, shown in Figure 3, a three way ANOVA revealed no significant main effect of outcome, *F*(1, 38) < 1, *p* = .94, but a significant interaction between outcome and group, *F*(1, 38) = 5.11, *MSE* = 2.11, *p* = .05, η_p_^2^ = 0.12 and between outcome and discrimination, *F*(1, 38) = 12.66, *MSE* = 1.36, *p* < .001, η_p_^2^ = 0.25. Paired samples *t* tests for the local group, showed no significant difference between initial judgments of BC and ABC, *t*(19) < 1, *p* = .86 or EF and GHI, *t*(19) = 1.93, *p* = .07. Showing an expected pattern of generalization on the basis of similarity, the global group judged ABC as more likely to be followed by the outcome than BC, *t*(19) = 3.10, *p* < .01, *d* = 0.85, 95% CI [0.24, 1.43], but did not give significantly different judgments for EF compared to GHI, *t*(19) = 1.18, *p* = .25. The relatively strong judgments following ABC at the start of training are expected given the similarity between the compounds in the negative patterning discrimination. The difference in judgments is in the opposite direction to the training contingencies and therefore could not be seen to contribute to the final discrimination.

On the last trial block of training, shown in Figure 3, a three way ANOVA revealed a significant three way interaction between outcome, discrimination, and group, *F*(1, 38) = 7.01, *MSE* = 1.65, *p* < .01, η_p_^2^ = 0.16. In the linear discrimination there was no significant interaction between outcome and group, *F*(1, 38) < 1, *p* = .88. All participants judged GHI (7.49, *SEM* = 0.21) as more likely to be followed by the outcome than EF (2.36; *SEM* = 0.19), *t*(39) = 19.92, *p* < .001, *d* > 2. In the negative patterning discrimination, there was a significant interaction between stimulus and group, *F*(1, 38) = 5.85, *MSE* = 3.63, *p* < .05, η_p_^2^ = 0.13. Paired samples *t* tests revealed that on the final trial block of training, the global group judged the outcome to be significantly more likely to follow BC than ABC, *t*(19) = 6.89, *p* < .001, *d* > 2. In contrast, for the local group, judgments of outcome likelihood following BC were not significantly higher than judgments following ABC, *t*(19) = 1.99, *p* = .06. This analysis indicates that on the final trial block of training both groups were discriminating between EF and GHI in the linear discrimination. In contrast, only the global group learned to discriminate between BC and ABC in the negative patterning discrimination.

The previous analysis divided participants into local and global groups to allow detailed exploration of learning. We also look at the relationship between discrimination and global processing as a continuous variable. To do this discrimination difference scores were calculated to give a single measure of discrimination learning. This was used to assess the proportion of variance in discrimination learning that was accounted for by the global processing score. A linear and a negative patterning discrimination score was calculated for the first trial block and for the last trial block of training. The linear discrimination score was calculated as the judgment of outcome likelihood following GHI minus the judgment following EF. The negative patterning discrimination score was calculated as the judgment of outcome likelihood following BC minus the judgment following ABC. Discrimination scores for the first trial block of training were then subtracted from discrimination scores on the last trial block of training to give an indication of the magnitude of change in discrimination over training.

**Figure 4 fig4:**
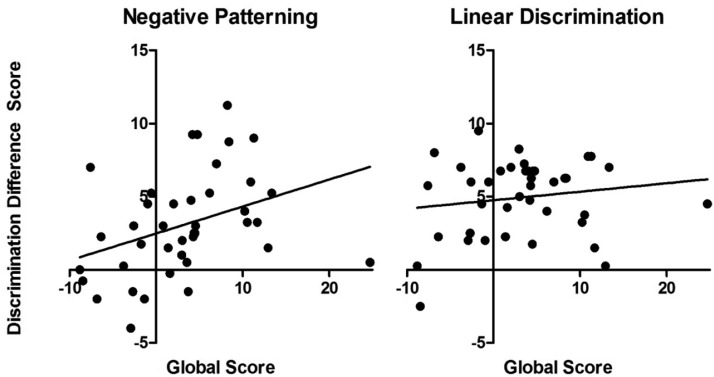
The relationship between discrimination difference score and global score for the negative patterning (left) and linear (right) discriminations in Experiment 1, showing line of best fit.

The relationship between global processing score and discrimination difference score for the negative patterning and linear discrimination is shown in Figure 4. The global processing score significantly predicted acquisition of the negative patterning discrimination, β = 0.36, *t*(38) = 2.40, *p* = .05, 95% CI [0.03, 0.34], and explained a significant proportion of variance in acquisition of the negative patterning discrimination, *R*^2^ = 0.13, *F*(1, 38) = 5.74, *p* = .05. Global processing score did not predict acquisition of the linear discrimination, β = 0.15, *t*(38) < 1, *p* = .34, or explain a significant proportion of variance, *R*^2^ = 0.02, *F*(1, 38) < 1, *p* = .34. Including digit span in the model did not increase the proportion of variance in acquisition of the negative patterning discrimination explained, *R*^2^change = 0.02, *F*(1, 37) < 1, *p* = .38 or increase the proportion of variance in acquisition of the linear discrimination explained, *R*^2^change = 0.01, *F*(1, 37) < 1, *p* = .57. Digit span was not correlated with global processing score, *r*(40) < .01, *p* = .98.[Fig-anchor fig4]

Overall, the results demonstrate considerable individual difference in ability to solve a negative patterning discrimination, in absolute terms and relative to the limited individual difference in ability to solve a linear discrimination. This does not appear to be explained simply in terms of working memory capacity. Global processing was correlated with ability to solve the negative patterning discrimination.

## Experiment 2

In Experiment 2 we asked whether the relationship between global processing and configural learning is static, or whether short term experiences can have a similar influence on ability to learn a configural discrimination. Experience identifying global targets in the Navon task has been found to enhance recognition of faces (Gao, Flevaris, Robertson, & Bentin, 2011; Macrae & Lewis, 2002; Perfect, 2003). Facial recognition is acknowledged to be dependent on global or configural processing (Bartlett & Searcy, 1993; Diamond & Carey, 1986; Leder & Bruce, 1998, 2000; Maurer et al., 2002; Tanaka & Farah, 1993; Tanaka, Kiefer, & Bukach, 2004; Tanaka & Sengco, 1997; Young, Hellawell, & Hay, 1987). Providing experience identifying local targets impairs the recognition of faces (Macrae & Lewis, 2002; Perfect, 2003) but enhances recognition of features (Weston & Perfect, 2005).

Given these findings we might anticipate that providing participants with experience identifying targets at one or other level may influence their tendency to process the elements of compounds or configurations and thereby influence discrimination learning. Therefore, experience identifying global targets may be expected to enhance a participant’s ability to learn a negative patterning discrimination, while experience identifying local targets may be expected to impair ability to learn a negative patterning discrimination.

### Method

#### Participants

There were 40 university students who participated for course credit or were paid £5 for their participation. Twenty-four participants were female. The average participant age was 22.33 years (*SEM* = 0.50) and the average digit span was 7.76 digits (*SEM* = 0.09). Eighteen participants completed the local pretraining and 22 participants completed the global pretraining.

#### Stimuli and materials

All participants completed the learning task and the Navon task as described in Experiment 1.

#### Procedure

Participants completed the Navon training task prior to the learning task. The procedure for the Navon training was similar to that used in the Navon test in Experiment 1, except that participants in the global training condition were asked to identify the large letter on all trials while participants in the local training condition were asked to identify the small letter on all trials. The negative patterning discrimination was completed as described in Experiment 1.

### Results and Discussion

Following the method described in Experiment 1, individuals who were unable to solve the linear discrimination were removed from analysis. This excluded one participant from the local pretraining group. On average participants made 6.88 errors (*SEM* = 0.545) on the Navon task. Participants with an error rate more than two times the standard deviation above the mean were excluded. This excluded one participant from each pretraining group. Following these exclusions the groups did not differ in terms of age, *F*(1, 35) = 1.77, *p* = .19, gender, *F*(1, 35) < 1, *p* = .68, or working memory capacity, *F*(1, 35) < 1, *p* = .93.

**Figure 5 fig5:**
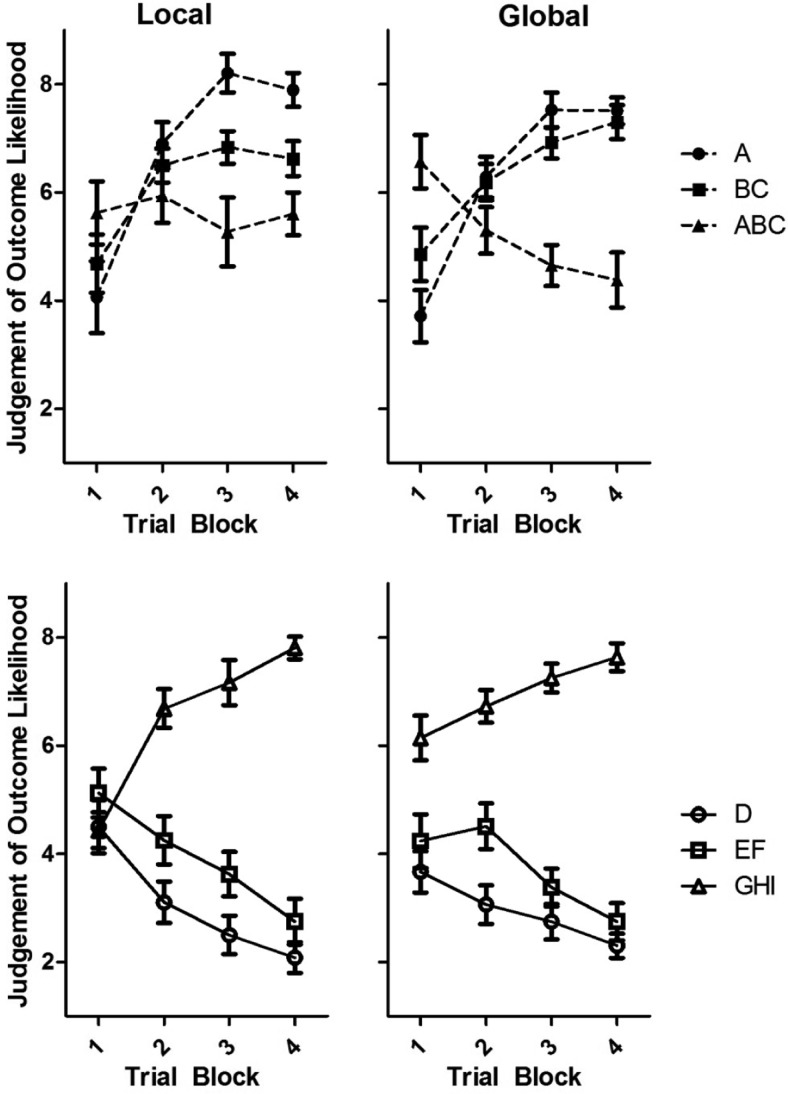
Judgments of outcome likelihood over trial blocks for Experiment 2, showing the local group (left) and the global group (right), for the negative patterning discrimination (top) and linear discrimination (bottom).

The analysis addresses whether the type of pretraining participants received on the Navon task influenced their discrimination learning. Judgments of outcome likelihood over training for the linear and negative patterning discrimination are shown in Figure 5. The global pretraining group showed a greater change in negative patterning discrimination over training than the local pretraining group. A four way repeated-measures ANOVA was conducted on judgments of outcome likelihood with the factors of discrimination (linear vs. negative patterning), outcome (cell growth vs. no cell growth), trial block (first vs. last), and pretraining group (local vs. global). This revealed a significant four way interaction, *F*(1, 35) = 15.24, *MSE* = 2.21, *p* < .01, η_p_^2^ = 0.30. To understand this interaction analyses of judgments of outcome likelihood on the first and final trial block were conducted, with the factors of discrimination (linear vs. negative patterning), outcome (cell growth vs. no cell growth), and pretraining group (local vs. global).[Fig-anchor fig5]

The analysis of the first trial block, using a three way repeated-measures ANOVA, revealed a significant interaction between discrimination and outcome, *F*(1, 35) = 7.08, *MSE* = 4.80, *p* < .01, η_p_^2^ = 0.17, and a significant interaction between discrimination, outcome, and pretraining group, *F*(1, 35) = 5.37, *MSE* = 4.80, *p* < .05, η_p_^2^ = 0.13. Paired samples *t* test for the local pretraining group showed no significant difference between initial judgments of BC and ABC, *t*(16) = 1.05, *p* = .31 or EF and GHI, *t*(16) = 0.98, *p* = .35. As observed in Experiment 1, the global group judged ABC as more likely to be followed by the outcome than BC, *t*(20) = 2.48, *p* < .05, *d* = 0.76, 95% CI [0.12, 1.39]. Unexpectedly, the global group also judged GHI as significantly more likely to be followed by the outcome than EF, *t*(20) = 3.95, *p* < .01, *d* = 0.91, 95% CI [0.37, 1.43].

A similar analysis of the last trial block of training revealed a significant three way interaction between outcome, discrimination, and pretraining group, *F*(1, 35) = 6.48, *MSE* = 1.51, *p* < .05, η_p_^2^ = 0.16. There was no interaction between outcome and group for the linear discrimination, *F*(1, 35) = < 1, *p* = .81; all participants judged GHI (7.71, *SEM* = 0.17) as more likely to be followed by outcome than EF (2.75, *SEM* = 0.26), *F*(1, 35) = 191, *MSE* = 2.35, *p* < .001, η_p_^2^ = 0.85. In contrast, for the negative patterning discrimination, there was a significant interaction between stimulus and pretraining group, *F*(1, 35) = 5.33, *MSE* = 3.07, *p* < .05, η_p_^2^ = 0.13. As shown in Figure 5, on the final trial block of training the global pretraining group judged the outcome to be significantly more likely to follow BC than ABC, *t*(20) = 5.11, *p* < .001, *d* = 1.52, 95% CI [0.76, 2.25]. The local pretraining group did not give significantly different judgments of outcome likelihood following BC than ABC, *t*(15) = 1.79, *p* = .09, *d* = 0.71, 95% CI [0.12, 1.51]. This analysis indicates that on the final trial block of training both pretraining groups were successfully discriminating between EF and GHI in the linear discrimination. In contrast the global pretraining group showed stronger discrimination between BC and ABC in the negative patterning discrimination than the local pretraining group.

Analysis of judgments of outcome likelihood on the final trial block of training demonstrated group differences in acquisition of the trained discriminations. Using judgments of outcome likelihood to calculate discrimination difference scores, as described in Experiment 1, supplementary analysis demonstrated that these group differences were consistent with a training effect. Analysis of discrimination difference scores, reflecting the change in discrimination between the first and last trial block of training, found differences between the two groups. A 2 × 2 ANOVA was conducted on discrimination difference scores with the factors of discrimination (linear vs. negative patterning) and pretraining group (local vs. global). Although there was no main effect of discrimination, *F*(1, 35) = 2.36, *p* = .13, there was a significant interaction between pretraining group and discrimination, *F*(1, 35) = 15.24, *MSE* = 8.82, *p* < .001, η_p_^2^ = 0.30. Participants receiving global pretraining showed a small difference in their ability to learn the two discriminations, *t*(20) = 2.20, *p* < .05, *d* = 0.44, 95% CI [0.02, 0.86], with marginally stronger discrimination learning over training for the negative patterning (4.64, *SEM* = 0.85) than linear (2.98, *SEM* = 0.77) discrimination. In contrast, participants receiving local pretraining showed stronger discrimination learning over training in the linear (5.75, *SEM* = 0.82) than the negative pattering (1.96, *SEM* = 1.01) discrimination, *t*(15) = 3.01, *p* < .01, *d* = 1.03, 95% CI [0.25, 1.78].

As shown in Figure 5, initial discrimination between the compounds in the linear discrimination was comparatively strong for the global pretraining group. It is thus conceivable that, in a direct comparison between linear learning and negative patterning learning, the global group might appear to show strong learning with the negative patterning discrimination because change in discrimination over training for the linear discrimination is limited, due to initial training trials rather than a consequence of the learning. Comparing discrimination difference scores to the value zero (no improvement in discrimination over training), as a baseline, somewhat avoids this limitation. Concurring with the analysis reported above, discrimination difference scores for the global pretraining group were significantly greater than zero for both the linear, *t*(20) = 3.89, *p* < .01, *d* = 1.20, 95% CI [0.48, 1.90] and negative patterning, *t*(20) = 5.45, *p* < .001, *d* = 1.68, 95% CI [0.87, 2.47] discrimination. For the local pretraining group, discrimination difference scores were significantly greater than zero for the linear discrimination, *t*(16) = 7.06, *p* < .001, *d* = 2.49, 95% CI [1.36, 3.60] but not for the negative patterning discrimination, *t*(16) = 1.93, *p* = .072.

Providing experience finding local or global targets influenced the ability to learn a negative patterning discrimination. Following experience identifying local stimuli, acquisition of the negative patterning discrimination was weaker than it was following experience identifying global stimuli. Pretraining experience specifically affected the participants’ ability to learn the negative patterning discrimination, in which participants had to learn to treat a combination of stimuli as distinct and predictive of a different outcome from its constituent elements.

This experiment did not assess change in an individual’s ability to acquire a configural discrimination. This was not directly assessed because we can only compare a group given configural training with one given elemental training. However, taken together with the results of Experiment 1, the findings suggest that both effects are at work. Experience attending to local stimuli may weaken acquisition whereas experience attending to global stimuli may strengthen acquisition of a negative patterning discrimination. It seems clear that Experiment 2 altered learning of at least one of the discriminations. Although far from conclusive the mean discrimination difference scores in the negative patterning discrimination suggest that local and global pretraining had an effect in Experiment 2. The average negative patterning discrimination difference score in Experiment 1 was 3.08 (*SEM* = 0.56). In Experiment 2 the average negative patterning discrimination difference score following experience identifying local stimuli was less than this (1.96, *SEM* = 1.01) and greater than this following experience identifying global stimuli (4.64, *SEM* = 0.85). Further research, looking at change in ability to acquire discriminations, is necessary fully to understand the direction of these effects.

## General Discussion

This study investigated the relationship between global processing and individual differences in configural learning. Despite keeping the perceptual properties of the stimuli constant, considerable variation in ability to learn a negative patterning discrimination was observed. The negative patterning discrimination requires individuals to learn that the co-occurrence of stimuli predicts a different outcome to that predicted by any of the components independently. The tendency to focus on global stimuli contributed to variance in ability to learn this discrimination. Experience identifying global stimuli had a similar influence upon learning. These results suggest that focusing on global stimuli might facilitate a configural learning strategy relative to focusing on local stimuli. The distinction between focusing on global or local stimuli may reflect breadth in sampling capacity with a focus on global stimuli reflecting a broader sampling capacity.

The finding of individual differences in learning a negative patterning discrimination is consistent with previous research (e.g., Shanks & Darby, 1998) and the results expand upon observations of variability in linear associative learning (Kaufman, DeYoung, Gray, Brown, & Mackintosh, 2009). The studies presented here suggest that there may be considerably more variability in configural learning than linear learning. Experiment 2 demonstrated just how variable learning might be, as the hierarchal arrangement of information used in the pretraining phase of Experiment 2 is pervasive in the world around us.

The findings presented here have particular relevance for processes of learning in psychopathology or conditions of high stress. For instance, states of negative arousal have been shown to constrict breadth of attention with a shift toward local processing (Basso et al., 1996; Fredrickson & Branigan, 2005; Gasper & Clore, 2002). If local processing is associated with these conditions, learning may be constrained to elemental processing with reduce capacity to learn complex, configural relations. In relation to risk factors for psychopathology, further research, using direct measures of stress and anxiety, or manipulations to induce stress, may provide further insight into the relationship between attention and learning in risk for psychopathology.

The results presented here add further support to the argument that learning can vary between configural and elemental strategies. Previous accounts of flexibility in learning have focused on the influence of the perceptual properties of stimuli. For instance the replacement parameter *r* was introduced to REM learning to account for the perceptual interaction between stimuli of the same modality (Wagner, 2003). REM conceives of stimuli as represented by multiple elements. Representations for some of these elements are context independent and they are activated whenever the stimulus is present. Representations of other elements are context dependent, with elements being activated or inhibited depending on the combinations of stimuli presented (Brandon et al., 2000). For instance, when stimulus A is presented alone, representations of the elements A_1_ and A_2_ may be activated. When stimulus A is presented in combination with stimulus B, the element A_2_ may be replaced by a new element, A_3_. The replacement parameter *r* allows flexibility in the proportion of context dependent elements that are replaced (Wagner, 2003). The replacement parameter may account for the variation in discrimination learning observed here. For instance, a focus on global stimuli might be associated with a high *r* value. In this way a large proportion of context dependent elements would be replaced when the context in which stimuli were presented changed, for example, A compared to ABC.

Likewise, the discriminability parameter introduced by Kinder and Lachnit (2003) may account for the variation in the results observed here, if a focus on global stimuli were associated with high discriminability between stimuli and compounds. Specifically, the Kinder and Lachnit (2003) model might account for the individual difference observed here by suggesting that global processing reduced the perceived similarity between compounds.

These modifications introduce flexibility into both, an elemental model, in the case of the replacement parameter (Wagner, 2003) and a configural model, in the case of the discriminability parameter (Kinder & Lachnit, 2003). The modification to the elemental model rejects the possibility that configural theory provides a valuable contribution to understanding learning and instead develops a new model following elemental theory. The modification to Pearce’s configural model makes a similar rejection of the value of elemental theory. An alternative approach to account for flexibility is to acknowledge that both elemental and configural theory add value to our understanding of learning. Two learning strategies may be possible, such that under certain conditions learning is more likely to follow an elemental strategy, whereas under other conditions learning is more likely to follow a configural strategy (e.g., Fanselow, 1999; Williams et al., 1994).

Variation in absolute sampling capacity may provide a framework to account for flexibility between elemental and configural learning. Many contemporary models of associative learning assume sampling capacity to be limited, but do not consider whether this capacity might vary (e.g., McLaren & Mackintosh, 2000; Pearce, 1987, 1994). Individual difference in tendency to focus on specific details or global configurations may reflect variation in absolute sampling capacity. As an individual’s attention narrows to focus on specific details, he or she may sample fewer elements present on a trial. This might influence the individual’s capacity to engage in configural learning. Stimulus sampling theory (Estes, 1955) postulates that, on any given trial, only a subset of stimulus features are actually sampled. For example, with the co-occurrence of stimuli A, B, and C, though multiple features for each stimulus may be sampled (e.g., A_1_, A_2_, A_3_, etc.), only a subset will be sampled. To learn about and respond to the co-occurrence of stimuli as a distinct configuration (ABC), we might assume that features of each of the co-occurring stimuli must be sampled simultaneously (A_1_, B_2_, C_3_ or A_2,_ B_1_, C_2_, etc.). This assumption leads to the prediction that the probability of sampling a configuration, and thus being able to engage in configural learning, will decrease as the number of features sampled decreases. As such, broader sampling capacity, captured in these experiments by focusing on global configurations, may facilitate learning about configurations while a more limited sampling capacity may result in processing stimulus compounds elementally.

### Limitations

Throughout this paper we have considered configurations in a spatial sense. Alternatively it is possible to consider configurations in terms of conditional relationships (e.g., Grand & Honey, 2008). Further research will be necessary to test whether spatial relationships are important for the effects on configural learning observed here.

In addition to varying in the requirement for configural processing, the negative patterning and linear discrimination may be thought to vary along a continuum of difficulty. Various factors can contribute to the difficulty of a discrimination task. For instance, task difficulty may be expected to increase with the number of stimuli (e.g., Zentall, Jagielo, Jackson-Smith, & Urcuioli, 1987), the complexity of the stimuli (e.g., Honig & Thompson, 1982), the ratios of the different stimuli (e.g., Deisig, Lachnit, Giurfra, & Hellstern, 2001) and the similarity of compounds (e.g., Pearce, 1987). Though the negative patterning discrimination has a comparatively high degree of similarity between compounds, it presents participants with fewer stimuli than the linear discrimination.

Here, a participant’s ability to learn about configurations (tested through the negative patterning discrimination) was contrasted with learning a problem in which learning the configurations was not necessary to solve the discrimination (the linear discrimination). The similarity between compounds in these discriminations also varied. Similarity can be varied while maintaining the ability to solve a discrimination without learning about configurations, for instance both of the following discriminations can be solved without learning about the configurations, but the compounds in the second discrimination have a higher degree of similarity than the compounds in the first discrimination: (1) ABC+, ADE–; (2) ABC+, ABD–. Further exploration of individual difference in such designs is necessary to understand whether global processing always provides an advantage for learning in situations in which there is a high degree of similarity between compounds.

Task difficulty may also be expected to be influenced by the outcome that stimuli are paired with. Learning about the absence of an outcome has been observed to be slower than learning about the presence of an outcome (e.g., Livesey, Thorwart, & Harris, 2011). This may potentially slow down learning in the linear discrimination. We are not convinced however that participants in this experiment learned any less about the absence of cell growth than the presence of cell growth. In the linear discrimination average judgments of outcome likelihood for D and EF decreased over training by 3.28 (*SEM* = 0.29) units, while judgments of outcome likelihood for GHI increased by a similar magnitude (3.08, *SEM* = 0.29). The difference between these two changes was not significant, *t*(39) < 1.

Individuals differ in their ability to learn a negative patterning discrimination. Variation in ability to learn a negative patterning discrimination was observed here independent from variation in the perceptual properties of the stimuli. Variation, relating to individual tendency to focus on global configurations or local details may reflect a difference in adoption of a configural or elemental strategy. Change between strategies may be prompted by a change in the absolute number of elements sampled. The results presented here demonstrate considerable individual difference in learning and suggest that we need a better understanding of the factors that influence whether an elemental or configural learning strategy is adopted.
